# Characterization and Genomic Analysis of a Novel Jumbo Bacteriophage vB_StaM_SA1 Infecting *Staphylococcus aureus* With Two Lysins

**DOI:** 10.3389/fmicb.2022.856473

**Published:** 2022-04-28

**Authors:** Bingyan Zhang, Huzhi Sun, Feiyang Zhao, Qian Wang, Qiang Pan, Yigang Tong, Huiying Ren

**Affiliations:** ^1^College of Veterinary Medicine, Qingdao Agricultural University, Qingdao, China; ^2^College of Life Science and Technology, Beijing University of Chemical Technology, Beijing, China; ^3^Qingdao Phagepharm Bio-tech Co., Ltd., Qingdao, China

**Keywords:** *Staphylococcus aureus*, jumbo phage, biological characterization, genomic characterization, lysin, antibacterial activity

## Abstract

The development of new antimicrobial agents is critically needed due to the alarming increase in antibiotic resistance in bacterial pathogens. Phages have been widely considered as effective alternatives to antibiotics. A novel phage vB_StaM_SA1 (hereinafter as SA1) that can infect multiple *Staphylococcus* strains was isolated from untreated sewage of a pig farm, which belonged to *Myoviridae* family. At MOI of 0.1, the latent period of phage SA1 was 55 min, and the final titer reached about 10^9^ PFU/mL. The genome of phage SA1 was 260,727 bp, indicating that it can be classified as a jumbo phage. The genome of SA1 had 258 ORFs and a serine tRNA, while only 53 ORFs were annotated with functions. Phage SA1 contained a group of core genes that was characterized by multiple RNA polymerase subunits and also found in phiKZ-related jumbo phages. The phylogenetic tree showed that phage SA1 was a phiKZ-related phage and was closer to jumbo phages compared with *Staphylococcus* phages with small genome. Three proteins (lys4, lys210, and lys211) were predicted to be associated with lysins, and two proteins with lytic function were verified by recombinant expression and bacterial survival test. Both lys210 and lys211 possessed efficient bactericidal ability, and lys210 could lyse all test strains. The results show that phage SA1 and lys210/lys211 could be potentially used as antibiotic agents to treat *Staphylococcus* infection.

## Introduction

*Staphylococcus aureus* (*S. aureus*) is one of important zoonotic bacteria and poses a serious health risk to humans and livestock worldwide. The use of antibiotics can effectively curb the spread of *S. aureus*, but an increasing number of *S. aureus* strains have developed resistance to antibiotics. It is urgent to find new antibiotics or their alternatives (Medina and Pieper, 2016). Bacteriophages (phages) are a class of viruses that specifically infect bacteria. They are the most abundant biological entity on the plant with an estimated number of about 10^31^, which is about ten times greater than the number of bacteria (Jurczak-Kurek et al., 2016). Phages have been widely considered as effective alternatives to antibiotics (Wills et al., 2005; El-Gohary et al., 2014). Compared with lysogenic (temperate) phages, lytic phages are more likely to be used as antibacterial agents.

Lysins are cell wall-hydrolyzing enzymes that are encoded by phages and synthesized at the late stage of the replication cycle and can cause bacterial lysis and death (Schuch et al., 2013). A lysin is composed of catalytic domain (CD) and binding domain (BD). The binding domain can recognize the receptors on host cell wall and specifically lyse bacteria *in vitro*. Therefore, the lytic spectrum of lysins is wider than that of phages (Gutiérrez et al., 2018). As new antibacterial biological agents, lysins have various advantages, such as high efficiency, substrate specificity, resistance, and easy transformation (Loeffler et al., 2001; Nelson et al., 2001; Son et al., 2012). They are expected to become the next generation of antibacterial agents.

In general, phages with a genome size of less than 200 kb are classified as small-genome phages, and phages with a genome size of greater than 200 kb but less than 500 kb are classified as jumbo (giant) phages (Yuan and Gao, 2017). With the development of next-generation sequencing (NGS), many phages have been sequenced. At present, there are about 300 genome sequences of jumbo phages available in NCBI (Supplementary Table S1). Based on morphological characteristics, most jumbo phages belong to *Myoviridae*, and only 17 *Caulobacter* phages belong to *Siphoviridae* (Supplementary Table S1). So far, most of jumbo phages are isolated from Gram-negative bacteria, and only 11 phages are isolated from Gram-positive bacteria, including 7 *Bacillus* phages and 4 *Staphylococcus* phages (Uchiyama et al., 2014; Yuan and Gao, 2017; Lood et al., 2020). In addition, a lot of proteins encoded by jumbo phages are not well annotated. Therefore, it is important to unveil the function of these proteins and to understand the evolution of jumbo phages and their interaction with hosts.

## Materials and Methods

### Bacterial Strains and Culture Conditions

Bacterial strains used in this study are listed in Table 1. *Staphylococcus lentus* (*S. lentus*) JTB1-3 was used to isolate and produce phage SA1. All bacteria were cultured in LB medium (5 g/l yeast extract, 10 g/l tryptone, and 10 g/l NaCl) at 37°C.

**Table 1:** Lytic activity of phage SA1 and lys210 against various strains of *Staphylococcus*.

Strain	ST^a^	Spot test^b^	Relative EOP^c^	Lys210^b^	Isolation Source	Geographic Location
*S. aureus*	95	+	High	+	Human	Beijing, China
	239	−	NA	+	Human	Beijing, China
	1,607	+	Low	+	Human	Beijing, China
	5	−	NA	+	Human	Beijing, China
	7	+	High	+	Human	Beijing, China
	4,945	+	Low	+	Human	Beijing, China
	188	−	NA	+	Human	Beijing, China
	338	+	Low	+	Human	Liaoning, China
	6	−	−	+	Human	Beijing, China
	398	−	−	+	Human	Liaoning, China
	2,133	+	Low	+	Human	Beijing, China
	630	+	Low	+	Human	Beijing, China
*S. epidermidis*	262	+	Low	+	Human	Beijing, China
	152	+	Low	+	Human	Beijing, China
	59	−	−	+	Human	Beijing, China
	10	−	−	+	Human	Beijing, China
	213	+	Low	+	Human	Beijing, China
	483	+	Low	+	Human	Beijing, China
	844	−	−	+	Human	Beijing, China
	89	−	−	+	Human	Beijing, China
	133	+	−	+	Human	Beijing, China
*S. haemolyticus*	73	+	Low	+	Human	Beijing, China
	3	+	−	+	Human	Beijing, China
	49	+	High	+	Human	Beijing, China
	1	+	Low	+	Human	Beijing, China
	9	+	Low	+	Human	Beijing, China
	69	+	NA	+	Human	Beijing, China
	37	+	NA	+	Human	Beijing, China
	50	+	High	+	Human	Beijing, China
*S. lentus*	−	+	High	+	Swine	Shandong, China

a ST: sequence type.

b (+) and (−) represented that clear and no plaques could be observed after infection with phage or lys210, respectively.

c Relative EOP: The relative EOP was considered as “high” when the ratio difference between tested bacteria and host bacteria was > 50%. And < 50% was considered as “low.” NA = No activity.

### Isolation, Purification, and Sequencing of Phage SA1

Phage SA1 was isolated from untreated sewage of a pig farm in Shandong, China. *S. lentus* JTB1-3 was used to enrich phages directly from the sewage, and phages were isolated by the double-layer plate method (Zhao et al., 2019). Briefly, the sewage was filtered through a 0.22-μm filter (Millipore, United States) to remove bacteria and other particles. Then, 5 ml of the filtrate and 500 μl of *S. lentus* JTB1-3 were added to 50 ml of LB medium, followed by incubation at 37°C overnight with shaking. After centrifugation (12,000 g, 10 min) and filtration (0.22 μm, Millipore), 100 μl of the filtrate was mixed with 100 μl of *S. lentus* JTB1-3. The mixture was inoculated in soft agar (LB medium containing 0.7% agar) and poured on the surface of an agar plate (LB medium with 1.5% agar), followed by incubation at 37°C for 6 h. Phages were first purified by four successive single-plaque isolation and were further purified by polyethylene glycol (PEG)-NaCl precipitation and CsCl density gradient centrifugation (Yang et al., 2020).

For sequencing, genomic DNA (gDNA) was extracted using the phenol–chloroform method (Zhang et al., 2017). A library with an insert size of 200 bp was constructed using NEBNext Fast DNA Library Prep Set for Ion Torrent (New England Biolabs, United States). Sequencing was performed on a Miseq sequencer (Illumina, San Diego, CA, United States). The sequencing data were trimmed and assembled using Trimmomatic v0.36 and CLC Genomics Workbench (Qiagen Bioinformatics, Denmark), respectively.

### Characterization of Phage SA1

The morphology of phage SA1 was examined using transmission electron microscopy (TEM; Ackermann, 2009). Briefly, 30 μl of purified phage suspension was adsorbed on a copper grid for 15 min. After staining with 2% uranyl acetate for 10 min, the sample was examined using a JEM-1200 EX transmission electron microscope (JEOL, Tokyo, Japan) at an acceleration voltage of 100 kV.

The host range of phage SA1 was determined by plaque assay. Briefly, various strains of *Staphylococcus* (Table 1) were cultured at 37°C overnight, and 100 μl of each bacterial culture was mixed with 5 ml of LB-agar (0.7%, w/v) medium and poured on the LB-agar (1.5%, w/v) plate. After the top agar was solidified, 10 μl of phage filtrate was inoculated and cultured at 37°C overnight. The morphology and turbidity of plaques were used to evaluate the infectivity of phage SA1. In addition, the relative efficiency of plating (EOP) of the strains was tested. In short, 10 μl phage SA1 with different titers (10^0^–10^6^) was spotted onto the plate of different strains and incubated for 6 h at 37°C. The relative EOP value was determined by calculating the ratio of PFUs of each phage-susceptible strain to the PFUs obtained with *S. lentus* JTB1-3.

The life cycle of phages, including latent period, lysis period, and stable period, was determined by the one-step growth curve (Yang et al., 2019). Briefly, phage SA1 was mixed with *S. lentus* JTB1-3 at a MOI of 0.1 and incubated at 37°C for 5 min. After centrifugation (12,000 g, 30 s), the pellets were re-suspended in LB medium, followed by incubation at 37°C with shaking at 180 rpm. At specific time points, aliquots (100 μl) were taken and centrifuged (12,000 g, 30 s), and the titers of free phages in the supernatant were determined using the double-layer plate method. The experiments were repeated three times. The phage titer equals its dilution ratio times the number of spots on the plate times 10. The burst size was calculated as the ratio of the final count of liberated phage particles to the initial count of phage particles.

### Bacterial Challenge Assay

Three milliliters of host strain at the early exponential growth phase was taken out and inoculated into 30 ml LB medium; then, SA1 was infected with MOI of 1, 0.1, and 0.01, and the uninfected culture was used as negative control. After that, it required to take out 200 μl every hour and measure OD_600_ with multiskan FC Photometer (Thermo Fisher Scientific, United States) for 16 h.

### Proteomic Analysis

After CsCl gradient centrifugation, the phage was reduced with dithiothreitol (DTT, Promega, United States) and alkylated with iodoacetamide (IAM, Promega, United States). Then, the proteins were digested with trypsin overnight. After the termination of digestion with formic acid (FA, Dima Technology Inc.), the peptides were analyzed using an Ultimate 3000 HPLC system (Thermo Scientific, United States) coupled to a Q Exactive mass spectrometer (Thermo Scientific, USA). All predicted ORFs of phage SA1 were searched using MASCOT software (Matrix Science, Britain) at a significance threshold to *p* < 0.05.

### Genome Annotation and Bioinformatics Analysis

Genome annotation was carried out using RAST.^1^ All predicted ORFs were searched using BLASTp against the NCBI non-redundant protein database (nr) with an E-value cutoff of 1 × 10^−5^, and their putative functions were verified using HHpred (Altschul et al., 1990; Söding et al., 2005; Lavigne et al., 2008).^2^ Putative tRNA was predicted using tRNA-scan-SE.^3^ A phylogenetic tree was constructed using MEGA-X. The genomic termini was analyzed using PhageTerm (Garneau et al., 2017).

### *In silico* Analysis and Homology Modeling of Two Lysins

Based on BLASTp verification, the domains of ORF4 (lys4), ORF210 (lys210), and ORF211 (lys211) were predicted by Pfam database^4^ and Conserved Domains Database (CDD).^5^ Signal peptides and transmembrane region were predicted by SignalP^6^ and TMHMM,^7^ respectively. Homology models were built by Robetta Server,^8^ and the quality of the model was evaluated by ERRAT and VERIFY 3D of SAVE.^9^

### Expression and Antibacterial Activity of Two Lysins

Nucleotide sequences of ORF210 (lys210) and ORF211 (lys211) were amplified by PCR with primers listed in Table 2. The recombinant plasmids pET-28a-lys210 and pET-28a-lys211 were constructed using ClonExpress Ultra One Step Cloning Kit (Vazyme, China). The recombinant plasmids were verified by sequencing and transformed into *E. coli* BL21. Positive transformants were cultured in LB medium (containing 50 μg/ml kanamycin) at 37°C until the culture reached an OD_600_ of 0.6. After addition of 0.1 mM IPTG, the transformants were cultured at 16°C (lys210 at 20°C) for 16 h. The cells were harvested and re-suspended in Tris buffer (50 mm Tris–HCl, 500 mM NaCl, 10% glycerol, pH 7.4) and disrupted using a high-pressure homogenizer (Aitesen, China) at 1,300 bar. After centrifugation at 12,000 g for 1 h, proteins in the supernatant were purified using Ni-NTA affinity chromatography (Biorigin, China) and imidazole was removed by ultrafiltration using a 10-kDa cutoff membrane (Millipore, MA, United States). The molecular weight and concentration of proteins were determined by 15% SDS-PAGE and BCA Protein Quantification Kit (Yeasen, China), respectively. At the same time, the purified protein was identified by mass spectrometry. Meanwhile, these two purified proteins were identified by Q Exactive mass spectrometer (Thermo Scientific, United States). The antibacterial activities of two proteins (lys210 and lys211) against *S. lentus* JTB1-3 were determined by the bacterial survival test. Briefly, host JTB1-3 at the early exponential growth phase was washed with sterile water three times. Then, 10 μl of purified proteins was mixed with 90 μl of JTB1-3 bacterial suspensions (10^8^ CFU/ml) at a final concentration of 129 μg/ml, and the mixture was incubated at 37°C for 1 h. Five microliters of tenfold serially diluted cell suspension was spot-plated onto LB agar dishes. Residual viable cell numbers on the plate were measured after incubation at 37°C for 12 h. The same volume protein expressed by the pET28a plasmid was used as the control, and all experiments were performed in triplicate.

**Table 2:** Primers used to amplify ORF210 and ORF211.

Gene name	Sequence
Lys210-F	cagcaaatgggtcgcggatccATGAAAACTAAAACTCAAGCTTTGAATT
Lys210-R	gtggtggtggtggtgctcgagTTAACTAAACGTACCCCATGCAGA
Lys211-F	cagcaaatgggtcgcggatccATGGCTAAAAAACACATTGGTACTT
Lys211-R	gtggtggtggtggtgctcgagTTAACTAAATGTTCCCCATGCTGG

## Results and Discussion

### Isolation and Morphological Characterization of Phage SA1

**Figure 1:**
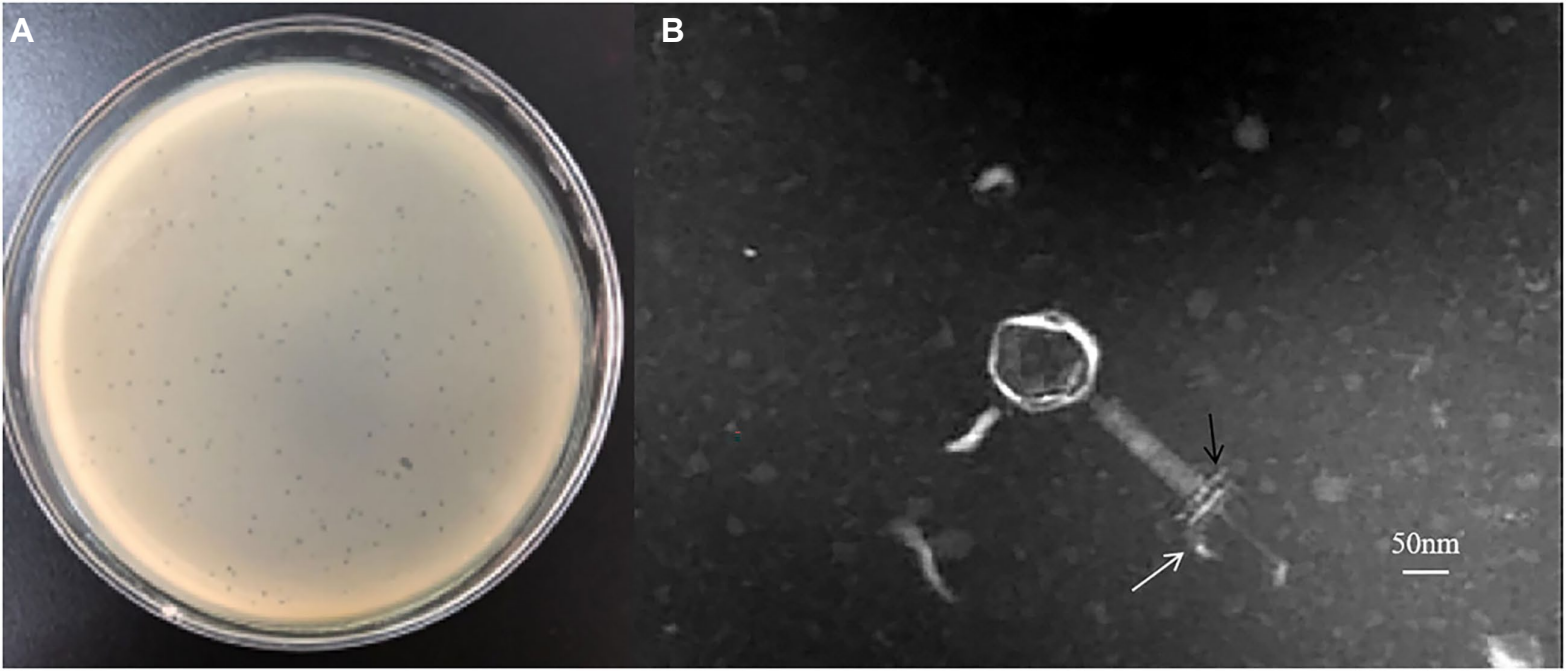
Isolation of a novel phage belonging to the *Myoviridae* family. **(A)** Plaques formed by phages SA1 on the lawn of host bacteria. **(B)** TEM image of phage SA1. The base-plate wedge and tail fibers are indicated by black and white arrows, respectively.

Phage SA1 was a lytic phage and could produce plaques of about 1 mm in diameter (Figure 1A). TEM images show that phage SA1 belonged to the *Myoviridae* family and possessed a polyhedral head (about 100 nm in diameter) and a long tail (about 220 nm in length). In addition, phage SA1 had a base plate and tail fibers (Figure 1B). Because jumbo phages are difficult to diffuse in soft agar, they usually cannot form visible plaques on double-layer agar plates, suggesting that it is difficult to isolate jumbo phages using the traditional double-layered plate method (Krylov et al., 2007). Compared with small-genome phages, jumbo phages might possess special structures. For example, *Bacillus* subtilis phage PBS 1 has several large helical tail fibers, which mediate the adsorption on the flagella of its host (Eiserling, 1967; Raimondo et al., 1968). And *Bacillus* phage vB_BpuM_BpSp has long baseplate-attached curly tail fibers (Yuan and Gao, 2016). In addition, the genomes of two *Pseudomonas aeruginosa* phages (EL and Lin 68) are packaged onto a spool-like protein structure called “the inner body,” which might be involved in DNA injection and packaging (Krylov et al., 1984; Sokolova et al., 2014).

### Host Range and One-Step Growth Curve of Phage SA1

The lytic activity of phage SA1 against 29 strains of *Staphylococcus* was tested, and the results are shown in Table 1. Besides the host *S. lentus*, phage SA1 could also infect *S. aureus* (7/12), *S. epidermidis* (5/9), and *S. haemolyticus* (8/8). To assess the infectivity of phage SA1 to different *Staphylococcus* strains, the relative EOP of phage SA1 was measured. It was found that SA1 could infect *S. aureus* (7/12), *S. epidermidis* (4/9), and *S. haemolyticus* (5/8). The differences in the results can be explained by the specificity of each method. In short, the wide host range indicates that phage SA1 has the potential to be used as an alternative to antibiotics in the treatment of *Staphylococcal* infection.

**Figure 2:**
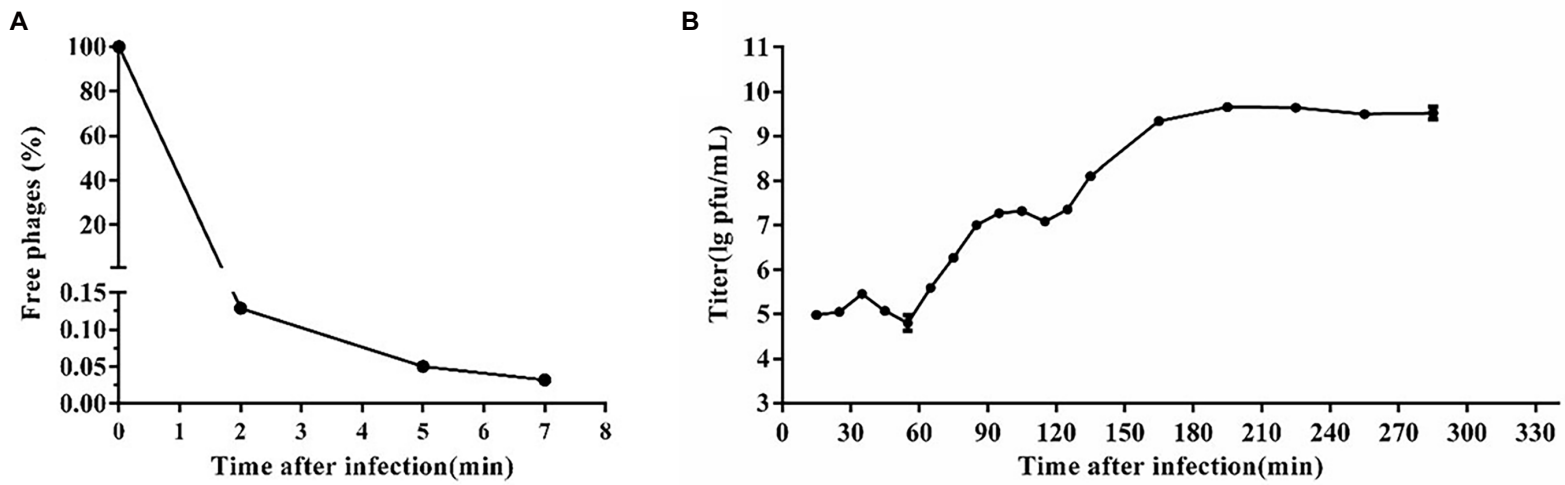
One-step growth curve of phage SA1. **(A)** Adsorption curve of phage SA1. Free phage (%) = the number of free phages after infection/the number of free phages without infection. After incubation for 5 min, free phages accounted for only 0.05% of phages in total. **(B)** One-step growth curve of phage SA1 after incubation for 5 min. The data are expressed as means ± SD (*n* = 3).

Based on the adsorption curve (Figure 2A), the one-step growth curve was outlined with incubating the phage SA1 with the host JTB1-3 for 5min. One-step growth curve shows that the latent period of phage SA1 was about 55 min, and the final titer reached about 10^9^ PFU/mL after two short burst periods (about 60 min and 50 min), with a burst size of about 130 and 140 PFUs/infected cell, respectively (Figure 2B). Multiple burst periods have been observed in other jumbo phages. *Staphylococcus* phage PALS2 is most similar to phage SA1 and had four short burst periods, with a burst size of 12 PFUs/infected cell after an additional latent period of 30 min and a final titer of up to 10^9^ PFU/mL (Lee et al., 2021). *Erwinia* jumbo phage Deimos-Minion has a latent period of about 3 h and double bursts of about 5 PFUs/infected cell (Sharma et al., 2019). The results suggest that jumbo phages can increase their production through multiple burst periods.

### Bacterial Challenge Assay

**Figure 3:**
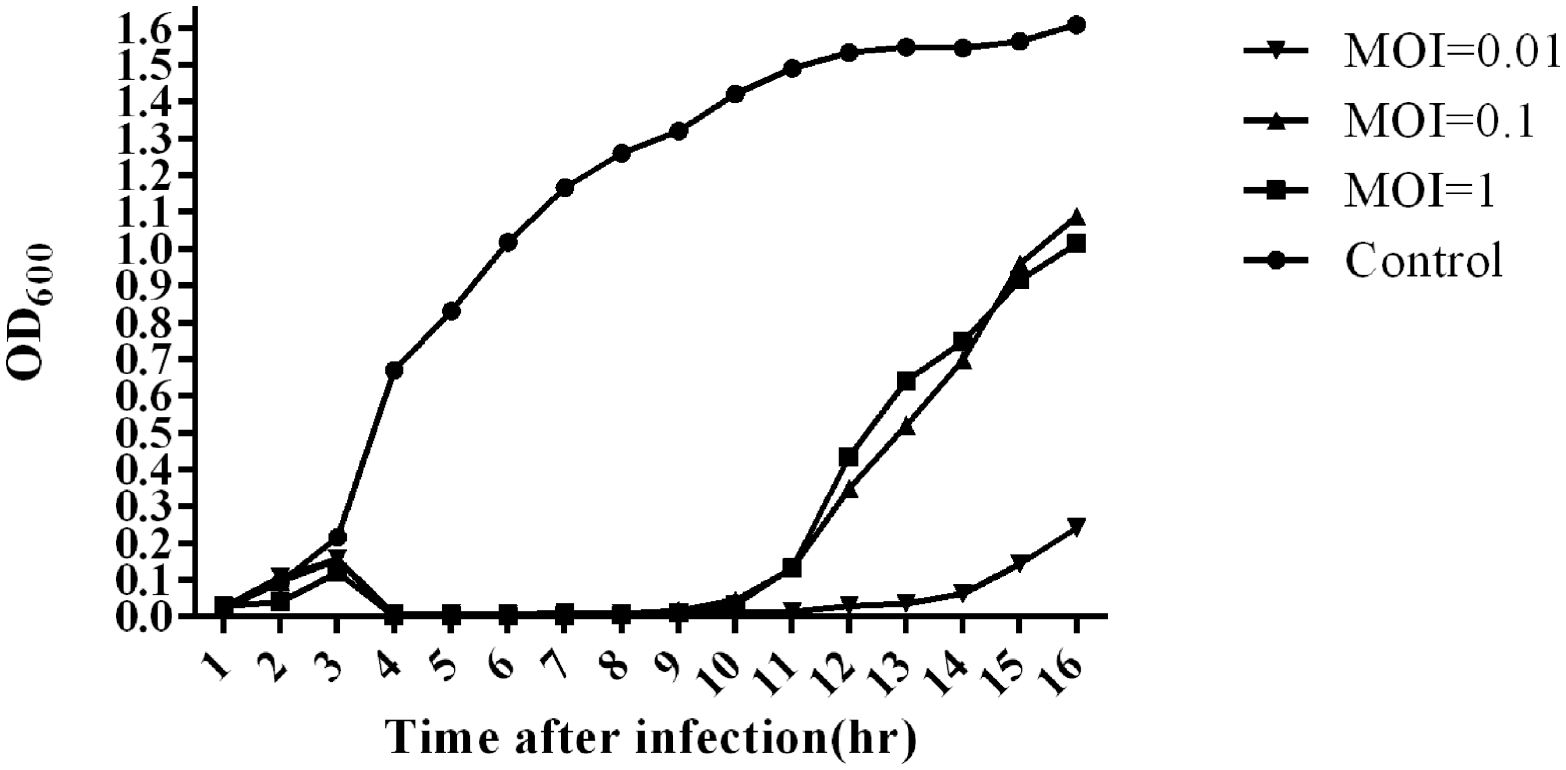
Bacterial reduction assay of phage SA1 against *S. lentus* JTB1-3 at various MOIs (1,0.1 and 0.01). The data are expressed as means ± SD (*n* = 3).

The bacterial challenge assay of SA1 on its host bacteria was tested by measuring the changes of OD_600_ after SA1 infection (Figure 3). SA1 was effective against the host strain in the tested MOIs, and almost no bacterial growth was observed in 10 consecutive hours after infection. In addition, when the MOI was 0.01, the bacteriostasis lasted up to 13 h, and the OD_600_ was less than 0.3 even at the 16th hour after infection. These results suggest that SA1 is a candidate for the treatment of *Staphylococcal* infection.

### General Genomic Features of Phage SA1

**Figure 4:**
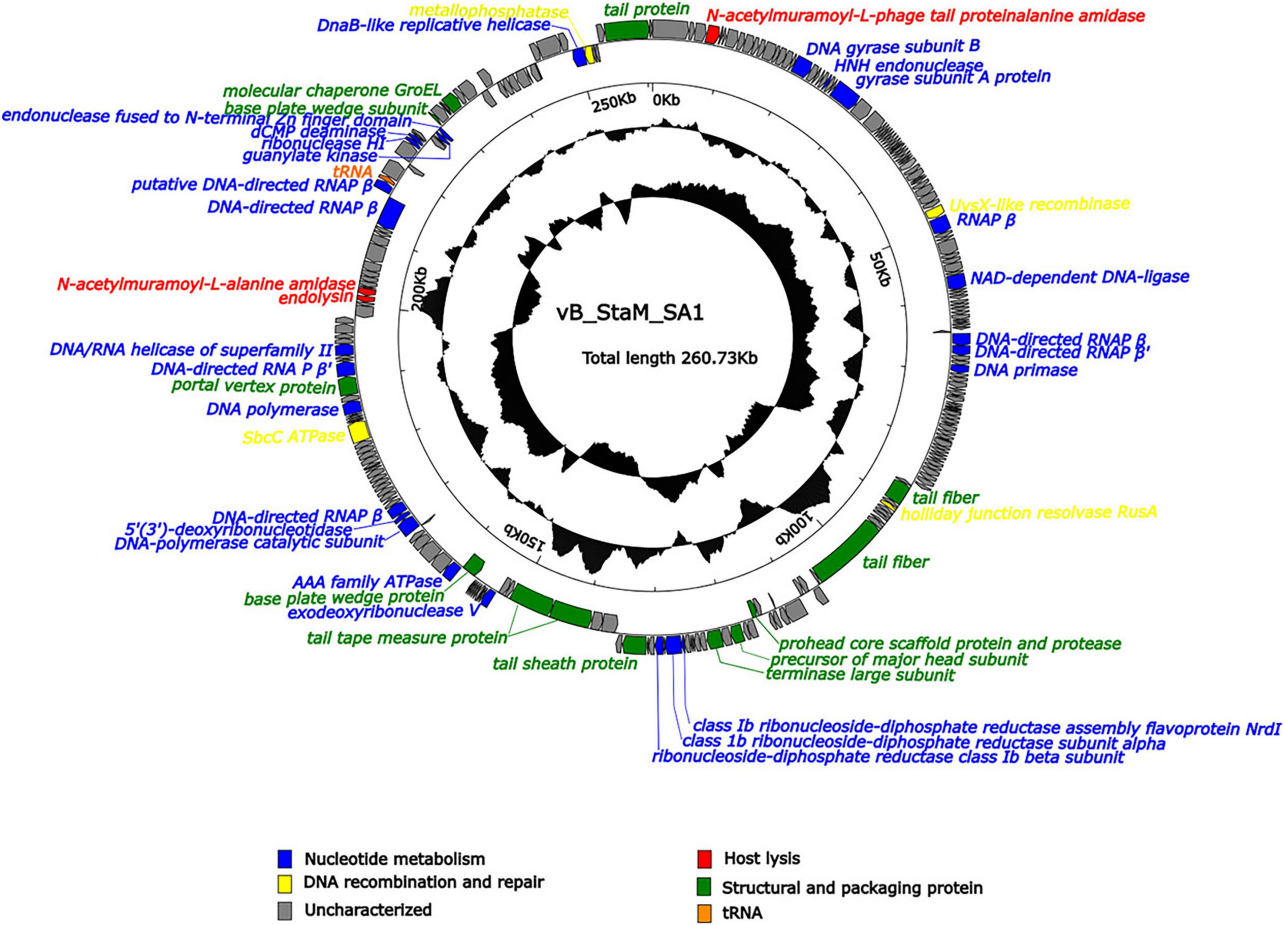
Arrangement of functional genes in the genome of phage SA1. The arrow represents the direction of transcription. Genes with different functions are represented in different colors.

Phage SA1 possessed a linear dsDNA genome of 260,727 bp, with G + C content of 26.83%, which was lower than that of the host (31.9%). The genome contained 258 predicted open-reading frames (ORFs) and a tRNA (ORF223, Ser), which could make up the deficiency of host tRNA in translation and expand the host range (Chen et al., 2018). Most of ORFs (78.4%) were located on the positive chain, with only 56 ORFs (21.6%) located on the negative chain, and the gene density was as high as 92.78% (Supplementary Table S2). There were no ORFs with predicted lysogenic function, such as transposase, integrase, and attachment site (attP), in the genome, indicating that SA1 was a lytic phage. Phage SA1 did not carry any virulence or pathogenicity genes, suggesting its potential application as an antibacterial and therapeutic agent. Terminal identification shows that SA1 had a headful packaging mechanism and belonged to P1-like phages. It means that, during the packaging process, the first cutting was conducted at the packaging site (PAC), and the subsequent cutting varied. BLASTp and CDD show that 53 ORFs were annotated to encode functional proteins (Figure 4), 47 ORFs shared homology with genes encoding hypothetical proteins in bacteria or phages, and 159 ORFs had no homology with the sequences in the database. The functional proteins were divided into four groups, including nucleotide metabolism (25 ORFs), DNA recombination and repair (4 ORFs), host lysis (4 ORFs), and structural and packaging proteins (14 ORFs; Figure 4).

### Analysis of Functional ORFs

Compared to small-genome phages, jumbo phages have more genes related to nucleotide metabolism (Hertveldt et al., 2005; Kiljunen et al., 2005; Thomas et al., 2007). Phage SA1 had 25 ORFs related to nucleotide metabolism, including 2 putative DNA polymerases (DNAPs) and 7 RNA polymerase (RNAP) subunits. Among 7 putative RNAP subunits, 6 had high identities (85.96% ~ 96.42%) with *S. aureus* jumbo phage PALS2 and 1 had high identities (91.33%) with *S. aureus* jumbo phage MarsHill. The core genes of phiKZ-related jumbo phage contained terminase large subunits (ORF138), DnaB-like replicative helicase (ORF254), and ribonuclease HI (ORF227), which could also be found in phage SA1 (Supplementary Table S2). In addition, ORF144, ORF145, and ORF147 of phage SA1 were predicted to encode alpha, beta, and NrdI subunits of ribonucleotide–diphosphate reductase (RNR) that could catalyze the biosynthesis of deoxyribonucleotides from the corresponding ribonucleotides to ensure a supply of precursors for phage DNA synthesis (Dwivedi et al., 2013). Having one or more RNAP subunits may be an important feature of jumbo phages. Similarly, *S. aureus* jumbo phage MarsHill, classified as phiKZ-related phages, has 10 RNAP subunits, including 4 β subunits and 6 β′ subunits (Korn et al., 2021). *Staphylococcus* phage PALS2 encoded 4 RNAP subunits, including β, β′, ω, and δ subunits (Lee et al., 2021). The presence of RNAPs in jumbo phages suggests that they reduced the dependence on the host and had more autonomy during gene regulation. For example, the presence of RNAPs has made the replication of *Pseudomonas* phage phiKZ independent of the host transcription apparatus (Ceyssens et al., 2014). Four ORFs related to DNA recombination and repairs were annotated in phage SA1. Among them, ORF192 (SbcC ATPase) and ORF255 (metallophosphatase) were supposed to form ATP-dependent dsDNA exonuclease repair complex SbcCD that could cleave hairpins in *S. aureus* (Chen et al., 2007). ORF53 (UvsX like recombinase) could bind to ssDNA or dsDNA to achieve error-free repair of DNA double-strand breaks by homologous recombination (Maher and Morrical, 2013). ORF116 was predicted to encode a holliday junction resolvase (RusA) that is a structure-specific endonuclease and could recognize holliday junction (HJ), cleave two similar DNA strands, and catalyze the occurrence of homologous recombination (Mahdi et al., 1996).

Three ORFs (ORF4, ORF210, and ORF211) encoded proteins associated with host lysis, whose amino acid sequence had 89.12, 80.94, and 85.22% identity to endolysin of *Staphylococcus* phage Madawaska, lysin of *Staphylococcus* phage PALS2, and N-acetylmuramoyl-L-alanine amidase of *Staphylococcus* phage MarsHill, respectively. However, we did not find a sequence similar to holin in NCBI. Holin is a class of proteins with 1–4 transmembrane helices and generally composed of 49–210 amino acids (Wang et al., 2000; Reddy and Saier, 2013; Savva et al., 2014). Therefore, 1 of 14 ORFs was supposed to encode holin-like protein, including ORF10, ORF24, ORF30, ORF38, ORF48, ORF84, ORF93-95, ORF117-119, ORF229, and ORF230. CDD results show that ORF117-encoded protein contained a holin_1 superfamily domain (cl02344) with an E value of 1.91e-08, so it was supposed to be holin because holin of *Staphylococcus* phage Machias has the highest identity of 64.94% with the protein encoded by ORF117 among these 14 ORFs (Korn et al., 2021).

In addition, the other 14 ORFs were predicted to encode structural and packaging proteins, including 3 (ORF133, ORF136, and ORF199) for head assembly, 8 (ORF114, ORF122, ORF150, ORF154, ORF155, ORF167, ORF235, and ORF252) for tail assembly, and 3 (ORF12, ORF138, and ORF239) for packaging. ORF199 (encoding portal vertex protein) might play an important role in head assembly, genome packaging, tail connection, genome excretion, and other morphogenetic processes (Isidro et al., 2004), and it could combine with ORF138 (encoding terminase large subunit) to form a packaging machine (Lebedev et al., 2007; Chang et al., 2012). ORF239 (encoding molecular chaperones) had a chaperonin-like superfamily domain (429 bp) and showed 34.41% identity with AR9. The GroEL-like chaperones of jumbo phages belong to group I chaperones, but they can exhibit activity without co-chaperones, which is different from the group I chaperones of eubacteria (Semenyuk et al., 2020).

Fifty-two proteins were validated by LC–MS/MS, including 11 virion-unrelated proteins, 34 gene products annotated as hypothetical proteins, and 7 virion-related structural proteins (Supplementary Table S2). Compared with small-genome phages, jumbo phages are considered to have more structural proteins. For example, *Pseudomonas* jumbo phages 201phi2-1 and phiKZ possess 89 and 62 structural proteins, respectively (Lecoutere et al., 2010; Thomas et al., 2010). However, some jumbo phages have only a few structural proteins, such as PALS2 that has only four structural proteins (Lee et al., 2021). There are only a few jumbo phage genomes in the database, which may be one of the reasons for this phenomenon. Therefore, isolating more jumbo phages and identifying phage-encoded proteins will contribute to the annotation and enhance our understanding of jumbo phages.

### Comparative Genomic and Evolutionary Analysis

**Figure 5:**
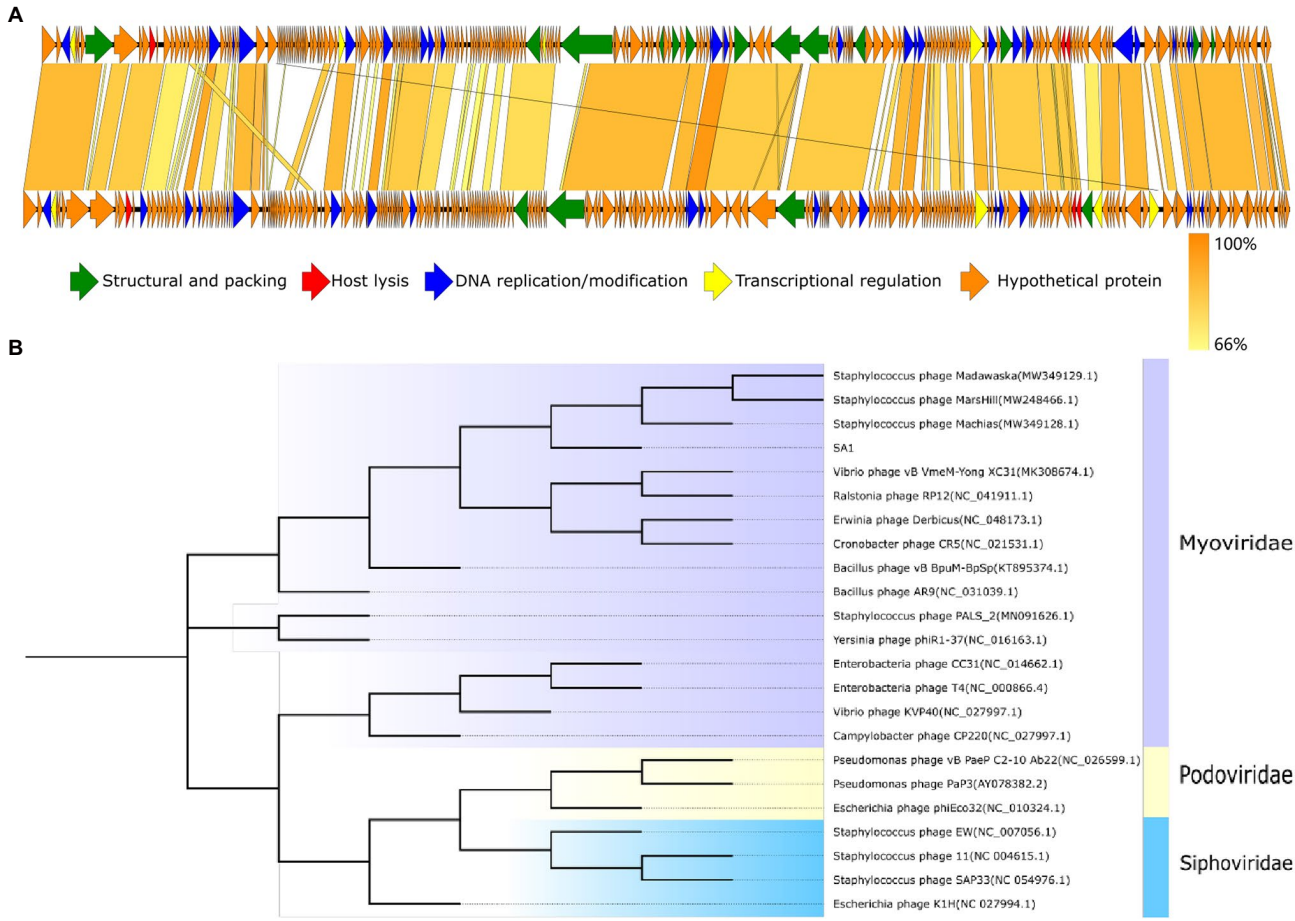
Evolutionary and comparative genome analysis of phage SA1. **(A)** Genome comparison between phage SA1(above) and PALS2(below). **(B)** Phylogenetic tree of phage SA1. The amino acid sequence of conserved protein terminase large subunit (ORF138) was aligned with the maximum likelihood method based on the JTT matrix-based model, with a bootstrap assessment based on 1,000 replicates. Genes with different functions in genome comparison and different subfamilies in the evolutionary tree are marked with different colors.

Phage SA1 shared the highest homology with *Staphylococcus* phage PALS2 in the database (Supplementary Table S3); then, two genomes were compared by multi-genome alignments. Results indicated that the functional genes of PALS2 and SA1 could be divided into the similar modules, and the functional genes of two phages were arranged similarly in the genome (Figure 5A). Sequence similarity of five phages were calculated by VIRIDIC^10^; results implied that the highest homology between them was 65.6%, which was lower than 70% of the threshold for classification as a genus. Therefore, we are proposing to create a new genus in the *Myoviridae* family to phage SA1.

To further analyze the genetic relationship between SA1 and other phages, amino acid sequences of conserved protein terminase large subunit were downloaded from NCBI database to construct a phylogenetic tree (Figure 5B). The phylogenetic tree shows that phage SA1 was closely related to jumbo phages, such as *Bacillus* phage AR9 (251,042 bp) and *Yersinia* phage phiR1-37(262,391 bp), but far from *Staphylococcus* phage 11(43,604 bp) and SAP33 (42,414 bp). The result indicates that, compared with *Staphylococcus* phages with a small genome, SA1 was closer to jumbo phages. Like MarsHill and PALS2, phage SA1 was supposed to be a member of phiKZ-like phages.

### *In silico* Analysis and Homology Modeling of Lys210 and Lys211

**Figure 6:**
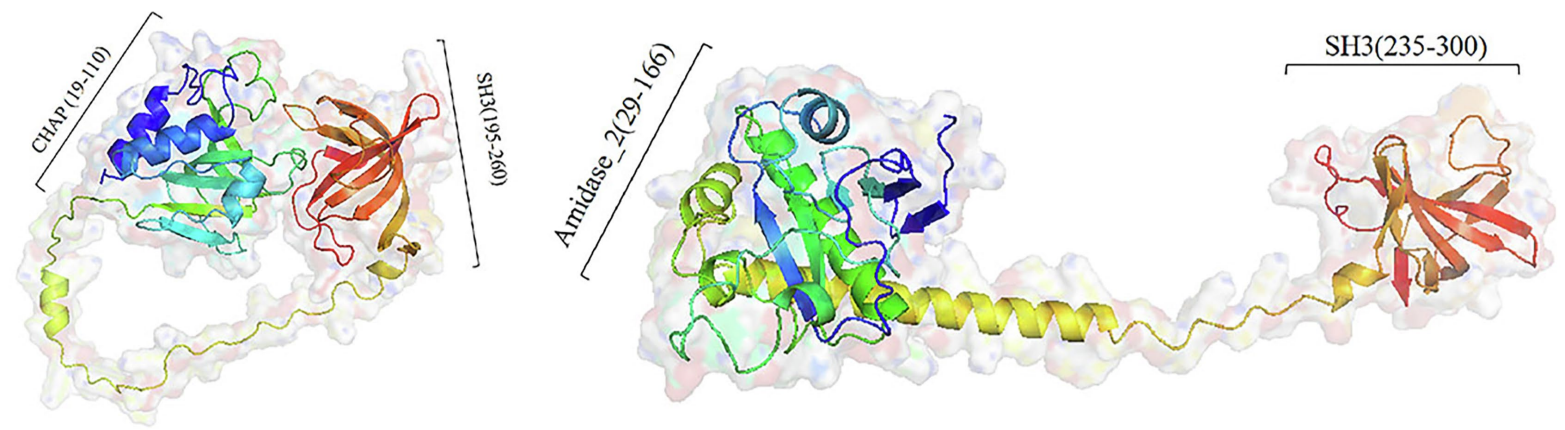
Homologous model structure of lys210 (left) and lys211 (right). Full-length amino acid sequences of lys210 and lys211 were used for 3D homology modeling by Robetta server and visualized using PyMol software.

All three predicted lysins (encoded by ORF4, ORF210, and ORF211) did not contain transmembrane segment and signal peptide, which was consistent with the general structure of lysin encoded by *Staphylococcus* phages (Rodríguez-Rubio et al., 2013). Domain analysis shows that ORF210 (lys210) contained a CHAP domain belonging to the NLPC_P60 superfamily (amino acids 19–110) and an SH3 domain (amino acids 195–260). And ORF211 (lys211) contained an amidase_2 domain (amino acids 29–166) and an SH3 domain (amino acids 235–300). In contrast, ORF4 (lys4) had only two N-terminal binding domains. The catalytic domains can cleave the bacterial cell wall, while the binding domains can only be anchored to the substrate, so we did not study the function of lys4. The full-length amino acid sequences of lys210 and lys211 were used for three-dimensional (3D) homology modeling. The online quality assessment shows that the 3D structures of lys210 and lys211 were credible because of two reasons: (1) more than 90% of the residues fell in favored and allowed regions (Ramachandran plot), and (2) the scores of lys210 and lys211 reached 89 and 96, respectively (Supplementary Figure S1). The predicted 3D structures of lys210 and lys211 clearly indicate that two distinct domains were connected by a linker (Figure 6). The SH3 domains of both lys210 and lys211 contained 7 β-strands, and the CHAP domain of lys210 contained 3 α-helices and 6 β-strands, and the CHAP domain of lys211 contained 4 α-helices and 6 β-strands (Figure 6).

It is reported that lysins with the catalytic domain of the NlpC/P60 superfamily protein have lytic activities (Mondal et al., 2021). The amidase_2 domain of lys211 showed zinc ion-dependent amidase activity. The truncated protein containing an amidase_2 domain of *Staphylococcus* phage 2638A could lyse *S. aureus* without zinc ions (Abaev et al., 2013). The lysin encoded by *Thermous* phage Ph2119 containing an amidase domain exhibits lytic activity against *thermophilic* bacteria and some Gram-positive and Gram-negative bacteria in the presence of zinc ions (Plotka et al., 2014). Therefore, lys210 containing CHAP domain and lys211 containing amidase_2 domain are considered to have lytic activity.

### Expression and Bactericidal Activity of Lys210 and Lys211

**Figure 7:**
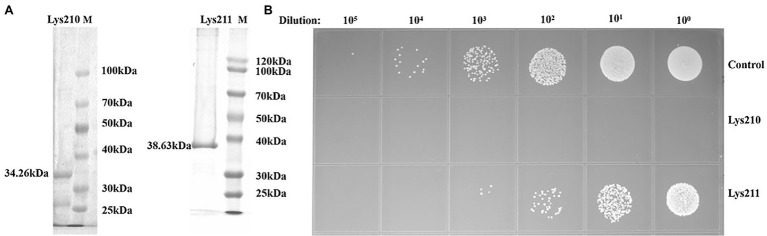
Antibacterial activity of lys210 and lys211. **(A)** Purification of proteins lys210 and lys211 expressed in *E. coli* BL21. The concentration of SDS-PAGE gel is 15%. **(B)** Survival test of *S. lentus* JTB1-3 on LB agar dishes after the cells were lysed by lys210 and lys211 (129 μg/ml) at 37°C for 1 h.

The recombinant proteins could be expressed in *E. coli* BL21, with molecular weights of about 34 kDa (lys210) and 38 kDa (lys211; Figure 7A). Through mass spectrometry identification, compared with the target protein sequences, the coverage of the two proteins reached 57 and 79%, respectively, and most of the peptide segments were unique (Supplementary Tables S4 and S5). Therefore, the obtained proteinS are the target proteins. At the final concentration of 129 μg/ml, lys210 could kill nearly all host cells, and lys211 also possessed efficient bactericidal activity (Figure 7B). In addition, lys210 could lyse all test strains, including *S. aureus* (12/12), *S. epidermidis* (9/9), and *S. haemolyticus* (8/8; Table 1). While lys211 did not show bactericidal activity on the plate, which could be due to the dry environment inhibited lys211 from exerting antibacterial activity.

Phage SA1 had a wide host range possibly because it could encode more active lysins. *Staphylococcus* jumbo phage PALS2 encoding 2 lysins (PALS2_239 and PALS2_240) can lyse various *Staphylococcal* strains, including *S. aureus, S. haemolyticus*, *S. hominis*, *S. warneri*, *S. saprophyticus*, *S. capitis*, *and S. cohnii* (Lee et al., 2021). Three *Staphylococcus* jumbo phages, including Madawaska (ORF152 and ORF202), Machias (ORF154 and ORF213), and MarsHill (ORF152 and ORF203), share similar genomic characteristics and are also annotated as two lysins, but their lytic spectrum and bactericidal activities of lysins remain unclear (Korn et al., 2021).

## Conclusion

In the present study, we identified a novel jumbo phage belonging to *Myoviridae* family, which can lyse various *Staphylococcal* strains. Genomic analysis shows that there are no virulence genes and pathogenic genes in SA1 meaning it could be used as a potential antibacterial agent in medical industry and animals. In addition, phage SA1 is a rare jumbo phage being isolated from Gram-positive bacteria, which enables us to better understand the diversity of Gram-positive jumbo phages and Gram-negative jumbo phages. The functions of two proteins predicted as lysin were tested. Both lys210 and lys211 show efficient bactericidal ability, and lys210 can lyse all test strains, indicating that lys210 and lys211 have promising characteristics for the development of biocontrol and detection tools.

## Data Availability Statement

The datasets presented in this study can be found in online repositories. The names of the repository/repositories and accession number(s) can be found at: https://www.ncbi.nlm.nih.gov/genbank/, MW218148.

## Author Contributions

BZ carried out the experiments, analyzed the data, and wrote this manuscript. HS and FZ performed bioinformatics analysis. QW isolated and characterized the phage. QP supervised the work. YT and HR designed the experiments and revised the manuscript. All authors read and approved the final manuscript.

## Funding

This research was supported by Donkey Industry Innovation Team Program of Modern Agricultural Technology System from Shandong Province, China (SDAIT-27), National Key Research and Development Program of China (NO. 2018YFA0903000, 2020YFC2005405, 2020YFA0712100, and 2020YFC0840805).

## Conflict of Interest

HS and QP were employed by the company Qingdao Phagepharm Bio-tech Co., Ltd.

The remaining authors declare that the research was conducted in the absence of any commercial or financial relationships that could be construed as a potential conflict of interest.

## Publisher’s Note

All claims expressed in this article are solely those of the authors and do not necessarily represent those of their affiliated organizations, or those of the publisher, the editors and the reviewers. Any product that may be evaluated in this article, or claim that may be made by its manufacturer, is not guaranteed or endorsed by the publisher.

## Supplementary Material

The Supplementary Material for this article can be found online at: https://www.frontiersin.org/articles/10.3389/fmicb.2022.856473/full#supplementary-material

## Footnotes

^1^ http://rast.nmpdr.org

^2^ https://toolkit.tuebingen.mpg.de/tools/hhpred

^3^ http://lowelab.ucsc.edu/tRNAscan-SE/

^4^ http://pfam.xfam.org/

^5^ https://www.ncbi.nlm.nih.gov/Structure/cdd/wrpsb.cgi

^6^ http://www.cbs.dtu.dk/services/SignalP/

^7^ http://www.cbs.dtu.dk/services/TMHMM/

^8^ http://robetta.bakerlab.org/

^9^ https://saves.mbi.ucla.edu/

^10^ http://viridic.icbm.de/
