# Supplementary figures and images for: Characterization and Genomic Analysis of a Novel Jumbo Bacteriophage vB_StaM_SA1 Infecting Staphylococcus aureus With Two Lysins
Source: Front Microbiol. 2022 Apr 28;13:856473. doi: 10.3389/fmicb.2022.856473 (PMC9096886; doi:10.3389/fmicb.2022.856473)

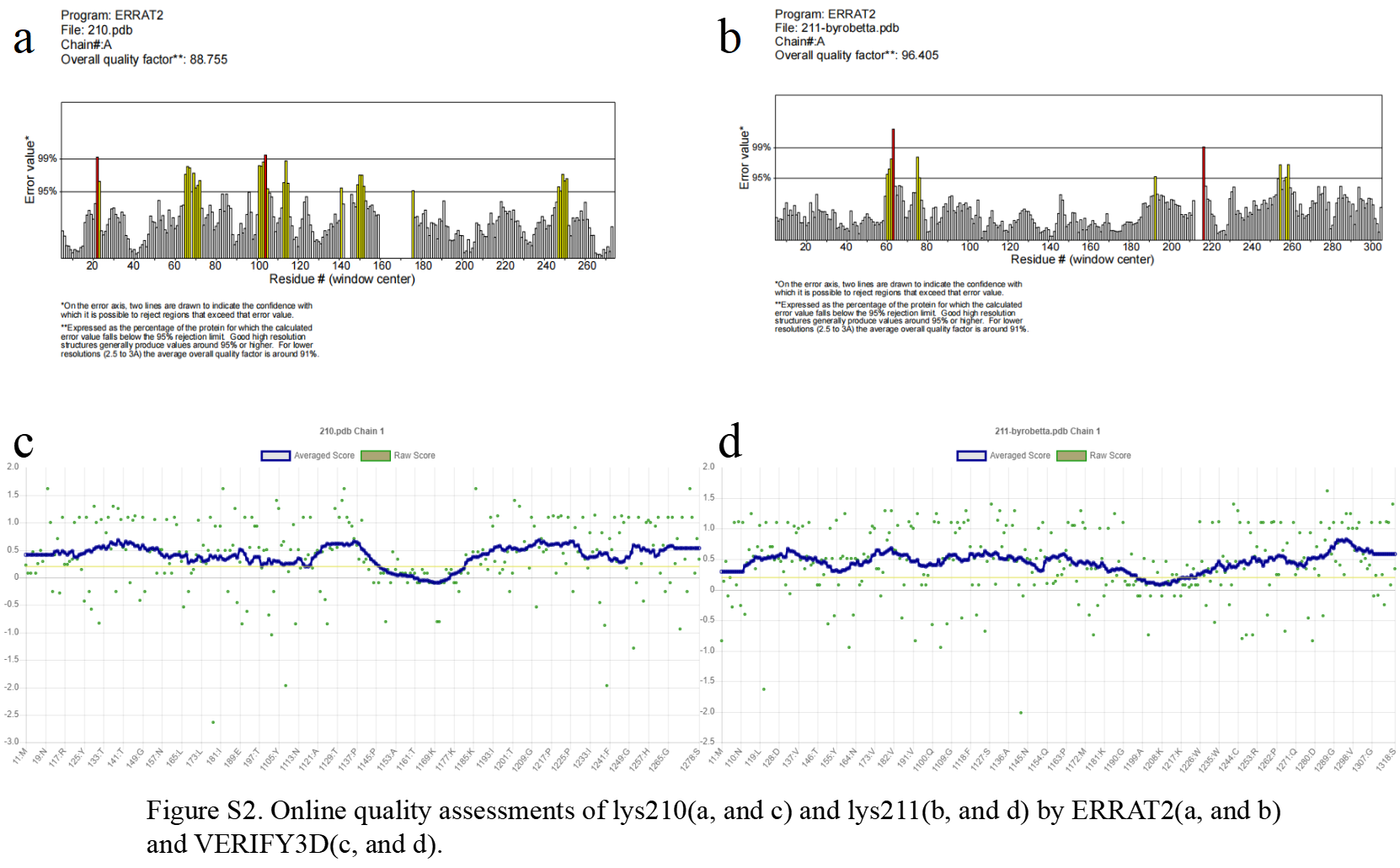

Supplement: Supplementary file 2 [file Image_1.PNG]
